# Thalamocortical Dysrhythmia: A Theoretical Update in Tinnitus

**DOI:** 10.3389/fneur.2015.00124

**Published:** 2015-06-09

**Authors:** Dirk De Ridder, Sven Vanneste, Berthold Langguth, Rodolfo Llinas

**Affiliations:** ^1^BRAI^2^N, Section of Neurosurgery, Department of Surgical Sciences, Dunedin School of Medicine, University of Otago, Dunedin, New Zealand; ^2^School of Behavioral and Brain Sciences, University of Texas at Dallas, Richardson, TX, USA; ^3^Department of Psychiatry and Psychotherapy, University of Regensburg, Regensburg, Germany; ^4^Department of Neuroscience and Physiology, New York University School of Medicine, New York, NY, USA

**Keywords:** thalamocortical dysrhythmia, theta, gamma, EEG, MEG, tinnitus, cross-frequency coupling, Bayes

## Abstract

Tinnitus is the perception of a sound in the absence of a corresponding external sound source. Pathophysiologically it has been attributed to bottom-up deafferentation and/or top-down noise-cancelling deficit. Both mechanisms are proposed to alter auditory ­thalamocortical signal transmission, resulting in thalamocortical dysrhythmia (TCD). In deafferentation, TCD is characterized by a slowing down of resting state alpha to theta activity associated with an increase in surrounding gamma activity, resulting in persisting cross-frequency coupling between theta and gamma activity. Theta burst-firing increases network synchrony and recruitment, a mechanism, which might enable long-range synchrony, which in turn could represent a means for finding the missing thalamocortical information and for gaining access to consciousness. Theta oscillations could function as a carrier wave to integrate the tinnitus-related focal auditory gamma activity in a consciousness enabling network, as envisioned by the global workspace model. This model suggests that focal activity in the brain does not reach consciousness, except if the focal activity becomes functionally coupled to a consciousness enabling network, aka the global workspace. In limited deafferentation, the missing information can be retrieved from the auditory cortical neighborhood, decreasing surround inhibition, resulting in TCD. When the deafferentation is too wide in bandwidth, it is hypothesized that the missing information is retrieved from theta-mediated parahippocampal auditory memory. This suggests that based on the amount of deafferentation TCD might change to parahippocampocortical persisting and thus pathological theta–gamma rhythm. From a Bayesian point of view, in which the brain is conceived as a prediction machine that updates its memory-based predictions through sensory updating, tinnitus is the result of a prediction error between the predicted and sensed auditory input. The decrease in sensory updating is reflected by decreased alpha activity and the prediction error results in theta–gamma and beta–gamma coupling. Thus, TCD can be considered as an adaptive mechanism to retrieve missing auditory input in tinnitus.

## Introduction

**Figure 1:**
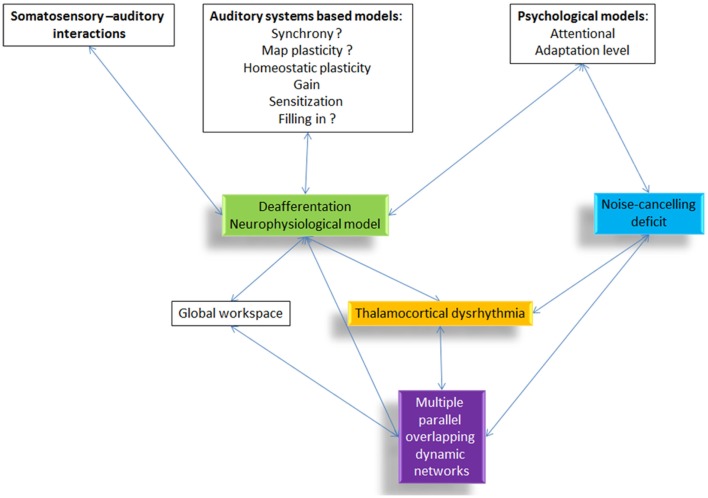
**Overview of the different tinnitus models and how they are related to each other**. All proposed pathophysiological models include or relate to thalamocortical dysrhythmia.

Non-pulsatile tinnitus is the perception of a sound in the absence of a corresponding external sound source. As an auditory phantom phenomenon, it has been conceptualized to be either linked to bottom-up deafferentation (see Figure 1, green box) with or without behaviorally measurable hearing loss, or a deficient top-down noise-cancelling mechanism (see Figure 1, blue box) or a combination of both.

Based on phenomenological similarities it has been proposed that tinnitus is the auditory analog of phantom pain (1–4) with auditory deafferentation representing its causative factor. Many subsequent models are refinements of this concept and limit themselves to changes in the auditory system, zooming in on the specific aspects linked to auditory deprivation, such as hyperactivity (5), an increase in gain (6), plasticity (7), including map plasticity (8) and homeostatic plasticity (9–11), synchrony (5, 12), filling the missing information (3, 13, 14), or sensitization (15). Many of these models are still a matter of debate, such as the involvement of synchrony (16), or whether changes in gain explain really tinnitus and not only hyperacusis. Also, the memory-based filling in mechanism is still a rather speculative model with limited experimental evidence. Moreover, some models focus on somatosensory compensatory mechanisms in the dorsal cochlear nucleus (17), which in itself has been proposed to be a core region in the development of tinnitus (18).

A second complementary concept proposes that deafferentiation-induced alterations in the central auditory pathways are not sufficient for the conscious perception of tinnitus. Tinnitus only arises, if auditory deafferentiation is accompanied by a deficient inhibitory top-down mechanism (19, 20), analogous to the anti-nociceptive system deficit linked to some forms of spontaneous pain such as fibromyalgia (21).

Recently, the concept has been forwarded that tinnitus might be the expression of a global workspace hyperactivity rather than being limited to the auditory system (22), which has been extended in a heuristic model integrating the deafferentiation model, the noise-cancelling deficit, and the global workspace model. The global workspace model was first proposed by Baars (23) as a model for conscious cognitive processing. It is a model for the interface between multiple unconscious, parallel processing modules on one side, and conscious experience on the other side, proposing how different sources of information are integrated into one percept with internally consistent content. The global workspace model suggests that multiple, highly specialized; quasi-independent input processors compete for access to a broadcasting capability by which the winning processor can disseminate its information globally throughout the brain. Based on the global workspace concept, Dehaene and colleagues (24) have proposed a neuronal implementation of a global workspace architecture, the so-called “neuronal global workspace.” In this model, sensory stimuli mobilize excitatory neurons with long-range cortico-cortical axons, leading to the genesis of a global activity pattern among workspace neurons, which are widely distributed throughout the brain. Any such global pattern can inhibit alternative activity patterns among workspace neurons, thus preventing the conscious processing of alternative stimuli and enabling a uniform conscious percept. Moreover, top-down attentional amplification is the mechanism by which modular processes can be temporarily mobilized and made available to the global workspace, and therefore to consciousness. This heuristic model is based on recent studies in consciousness research (25–27) and the philosophical concept of emergence in complex adaptive systems (3, 28–30). The emergence of a unified percept relies on the coordination of scattered mosaics of functionally specialized brain regions (31). This is proposed to be related to oscillatory activity and connectivity within the brain (32). Large-scale integration has been proposed to bind the distributed anatomical and functional organization of brain activity enabling the emergence of coherent behavior and cognition. Although the mechanisms involved in large-scale integration are still largely unknown, it has been convincingly argued that the most plausible candidate is the formation of dynamic links mediated by synchrony over multiple frequency bands (31). Even though within-frequency phase synchronization may support the binding of anatomically distributed processing, it cannot coordinate neuronal processing distributed into distinct time windows or frequency bands (33). Thus, cross-frequency coupling might be important for large-scale integration via low-frequency coherence of distributed geographically focal high-frequency activity (34). This could be carried out by nested oscillations (phase–amplitude interactions) or by phase–phase interactions, such as n:m phase synchrony, at discrete frequencies (33), as shown in both invasive human recordings (35) and with MEG and EEG recordings related to sensory awareness (36). This notion is supported by very recent results demonstrating tinnitus-related cross-frequency-coupling (37). Multiple forms of cross-frequency coupling exist. The most commonly studied forms are phase–amplitude and phase–phase cross-frequency coupling, but amplitude–amplitude, phase–frequency cross-frequency coupling exist (38). In tinnitus, intra-area cross-frequency coupling is known as thalamocortical dysrhythmia (TCD), and inter-area phase–amplitude coupling between anterior cingulate and auditory cortex and dorsolateral prefrontal cortex has been shown as well (37). The other cross-frequency couplings have not been investigated yet.

What all models, the deafferentation neurophysiological model, the noise-cancelling deficit, and the multiple parallel overlapping dynamical network model have in common, is that they rely on a final common pathway called TCD (39) (see Figure 1, yellow box). However, the question arises, how to theoretically integrate TCD in the setting of consciousness as an emergent property of network activity (31, 40) and more specifically in tinnitus (41).

Tinnitus is perceived as a unified percept, incorporating a sound, which can be different in quality, location, loudness, constancy, duration, as well as affective components such as mood and distress. It is assumed that each of these characteristics is related to a separate subnetwork, but linked by hubs that are part of multiple subnetworks (41). Furthermore, there are common associated symptoms in tinnitus, such as hearing loss, which can also generate distress (42, 43), and as distress is generated by a non-specific brain network (3, 44–49) this can be perceived or confounded as tinnitus distress. The lateralization of the tinnitus percept depends on gamma-band activity in the parahippocampal area, associated with functional connectivity between the parahippocampal area and auditory cortex.

Tinnitus has been likened to pain, both pathophysiologically, clinically, and treatment-wise (1–3, 39, 50–52). However, whereas different subtypes of pain have been clearly discerned, e.g., nociceptive versus neuropathic pain, this has not been as obvious for tinnitus. It is likely that different forms or subtypes of tinnitus exist, each with a different pathophysiology, which could explain the interindividual differences seen in recent electrophysiological studies (53, 54).

## Gamma-Band Activity and Consciousness

**Figure 2:**
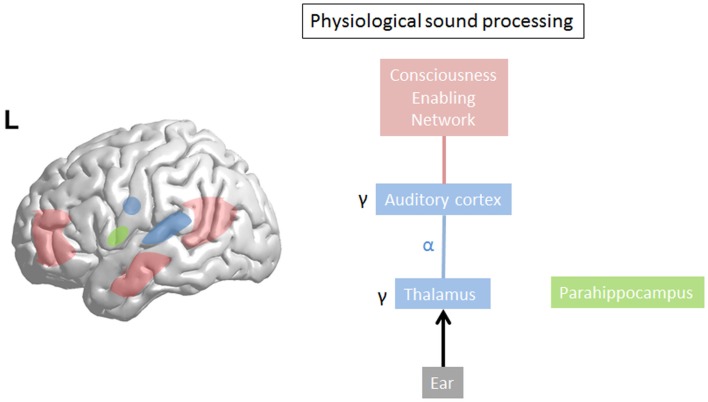
**In physiological sound processing, sound triggers gamma-band activity in the auditory cortex**. The externally presented sound only reaches consciousness if consciousness enabling network (=global workspace) is co-activated with sound perception. The last sound presentation is stored in the (para)hippocampal area as a reference for future input to calculate the prediction error.

Gamma-band activity (>30 Hz) is important for motor control (55) and processing of sensory stimuli, whether in the olfactory bulb (56), visual (57), auditory (58), or somatosensory cortex (59). Synchronization of separate gamma-band activities (>30), present in different thalamocortical columns (60), is proposed to bind (57, 61) distributed neural gamma activity into one coherent auditory percept (58, 62–66). Sound intensity is also reflected by the amount of gamma-band activity (67). This could suggest that gamma reflects a difference, a prediction error (68), a detection of change to the reference (69), or *status quo*, the latter, which would be reflected by beta activity (70). In general, gamma-band activity is present only in locally restricted areas of the cortex for short periods of time (34, 35, 71, 72). Whereas normally gamma activity waxes and wanes, related to the presence of a novel external stimulus, persisting gamma activity localized in one brain area has been considered pathological (39) (see Figure 2). However, in contrast to the waxing and waning of gamma-band activity, recent studies related to language processing do demonstrate there are also spontaneous endogenous longer lasting gamma rhythms present at rest in monkeys (73) and humans (74). These are likely generated by inhibitory interneurons (75) or interactions between pyramidal cells and interneurons (76, 77) and might reflect attentional processes (76, 77). These complement the exogenous stimulus driven gamma-band activity, which usually increases gamma-band activity in sensory areas, presumably clustering spiking activity that is propagated to higher hierarchic processing stages (74). Thus, it has been envisaged that these spontaneous gamma oscillations mechanistically might optimize the extraction of relevant sensory input in time (74). This intriguiging finding could potentially be applied to tinnitus. It has been proposed that oscillations correspond to the alternation of phases of high- and low-neuronal excitability, which temporally constrain sensory processing (78). This means that gamma oscillations, which have a period of approximately 25 ms, provide a 10- to 15-ms window for integrating spectrotemporal information followed by a 10- to 15-ms window for propagating the output (74). However, because the average length of a phoneme is about 50 ms, a 10- to 15-ms window might be too short for integrating this information, but a phoneme can correctly be represented by three gamma cycles, which act as a three-bit code (74). Further studies should evaluate whether tinnitus could be represented by such a 3-bit gamma code. In the visual system, stimuli that reach consciousness and those which do not reach consciousness are characterized by a similar increase of local gamma oscillations in the visual cortex (79, 80). Thus, gamma-band activity, *per se*, is not related to conscious perception, but could be a condition *sine qua non*, an essential prerequisite, for conscious perception (30). This is further confirmed by animal studies. Animals lacking gamma-band thalamocortical activation, due to the absence of P/Q-type Ca^++^ channels [CaV2.1-null (i.e., _-1A KO)], lacks a cognitive response to sensory stimulation (81). Only when focal gamma activity is embedded in a larger network of long-range functional connectivity, this will lead to the emergence of a conscious percept (41) (see Figure 2).

## Thalamocortical Dysrhythmia and Tinnitus

Based on the principle that gamma-band activity is associated with the conscious percept of auditory stimuli (58, 66, 82), Llinas proposed that tinnitus, as a conscious percept, should be associated with persistent gamma-band activity in the auditory cortex (39) confirmed in latter Ca V2.1-null experiments (81). Indeed, it has been shown both by EEG (83), MEG (39, 84, 85), and intracranial recordings (46) that tinnitus is related to gamma-band activity in the auditory cortex and nested on theta activity (39, 46, 84). Whereas, initially, it was thought that the gamma-band activity was contralateral (83, 84), it has been shown that gamma-band activity is increased in both auditory cortices, even in unilateral tinnitus (86). The amount of gamma-band activity has been shown to be related to the subjectively perceived tinnitus loudness (83). A decrease in tinnitus loudness has been found to be associated with a decrease in gamma-band activity in the auditory cortex (87–89) and a worsening in tinnitus loudness with an increase in auditory cortex gamma-band activity (90), even if such a relationship has been called into question by a recent study (54). In this paper (54), gamma-band activity increases and decreases were noted associated with an increase in tinnitus loudness after residual inhibition or a decrease in loudness after residual excitation, respectively. This could reflect that gamma represents a homeostatic change in loudness *per se*, on the condition that the tinnitus state has become the reference, or it could potentially be attributed to other factors, such as associated hearing loss in these patients.

An explanation for the occurrence of this theta–gamma coupled activity in sensory deafferentation is provided by the concept of TCD (39, 85). This model states that, in the deafferented state, the dominant resting state alpha rhythm (8–12 Hz) decreases to theta (4–7 Hz) (39) band activity. Conceptually, this can be explained that the firing rate decreases when less information needs to be processed (91) due to the deafferentation, and that firing and oscillation rate are coupled at the thalamocortical level (85). As a result, GABA_A_-mediated lateral inhibition is reduced (85), inducing gamma (>30 Hz) band activity (39) surrounding the deafferented theta area, also known as the edge effect (85). Indeed, in tinnitus, a decrease in alpha power is associated with an increase in gamma power (92), and gamma goes together with theta activity (84), confirming the initial studies by Llinas et al. (39, 85). Other studies have since demonstrated the presence of both low- (delta or theta) and high-frequency activity in the auditory cortex of tinnitus patients (88, 89, 93, 94), and the coupled activity has also been confirmed on electrode recordings of implanted patients (46).

It was proposed that theta reflects the negative symptoms (hearing loss, hypoesthesia, …) and gamma the positive symptoms (tinnitus, pain, …) in diseases characterized by TCD (85, 95). Thus, the negative symptoms (e.g., hearing loss, somatosensory deprivation in amputation, …) are proposed to be linked to less information processing and therefore slowed alpha activity, as if the deafferented thalamocortical columns are “as asleep” (85). It has been proposed that this theta could then act as a long-range carrier wave (96–98) on which the tinnitus information can be nested by means of high-frequency oscillatory activity (41). This notion has been confirmed by a MEG study, which demonstrated increased coupled gamma–theta wave activity in tinnitus patients (39, 84, 85) and recently demonstrated in EEG as well (99).

How is this TCD generated at a cellular level and what mechanism can link TCD to a more widespread phenomenon in the brain?

## T-Type Ca Channels, Theta Activity, Synchrony, and Network Recruitment in Thalamocortical Dysrhythmia

It has been proposed that the tectal system, with its rapid multisensory input, organizes the instantaneous orienting responses crucial to the maintenance of life (95). By contrast, the thalamocortical system, with its extensive corticothalamic recurrence and temporal binding properties, creates the complex combinatorial sensorimotor activities required for intentional behavior, characteristic of vertebrate behavior (95). Thalamic neurons, on being depolarized from resting potential levels positive to −55 mV, fire tonically. Close to the threshold level, gamma dendritic subthreshold oscillations occur supported by P/Q-type Ca channels (81). At hyperpolarized levels negative to −60 mV, de-inactivation of a Ca^2+^ conductance give rise to an inward current through T-type Ca channels, resulting in low-threshold burst firing. This remains for as long as the cells remain hyperpolarized, i.e., deafferented. Thus, in short, thalamic neurons have the unique property to fire both at depolarization and at hyperpolarization.

T-type calcium channels are known as low-voltage-activated (LVA) channels as they first open at more hyperpolarized membrane potentials compared to high-voltage-activated calcium channels, such as the L-, P/Q-, N-, and R-type channels (100). Thus, T-type calcium channels can be considered first-responders to small depolarization (100), generating a low-threshold Ca^2+^ potential upon the crest of which sodium and potassium channel-mediated action potentials fire, creating low-threshold bursts (101). Overall, the entire burst process mediated via T-type Ca channels takes approximately 100–200 ms limiting the frequency at which bursting can occur to around 5–10 Hz (102), i.e., theta frequencies. When a burst is generated in a thalamocortical neuron anywhere on the dendrite or cell body, the low-threshold Ca^2+^ potential is conducted not only to the soma but also back-propagates throughout the entire dendritic tree (100), generating bursts in multiple postsynaptic neurons in the projected nucleus through axonal arborization to multiple synapses. These bursting neurons will in turn recruit additional neurons in the reciprocally connected initiating nucleus and this burst amplification will progress on each cycle of the oscillation, generating synchronization in a network of reciprocally connected CNS nuclei (103, 104). This is in agreement with clinical data demonstrating that the dysrhythmia can be recorded with MEG over widespread areas (39). But the burst-firing process may also act as a neuronal frequency filter, preventing the conduction of recent incoming postsynaptic activity to the soma, and thereby preventing other activities contributing to the neuronal output (99).

In summary, in the non-bursting regions subthreshold oscillations are capable of performing a filtering task without increasing neuronal output. Contrastingly, bursting regions may spread network oscillations and recruit interconnected cortical areas, while simultaneously acting as a theta frequency filter (100). As such theta burst-firing increases network synchrony and recruitment (100), a mechanism, which might enable long-range synchrony, which in turn could represent a means for finding the missing thalamocortical information (due to deafferentation) (105). Hypothetically, the missing information may be replaced from (para)hippocampal memory. The synchrony would permit the (para)hippocampal information to be boosted to conscious perception by recruiting consciousness enabling network areas (41).

## Thalamocortical Dysrhythmia Versus Physiological Cross-Frequency Coupling

Gamma-band activity in the auditory cortex is a prerequisite for auditory conscious perception and, therefore, likely also contributes to the perception of a phantom sound (Table 1). As theta–gamma coupling also exists in physiological auditory processing (34, 35, 106), the TCD state can be considered as a pathological persistence of normally waxing and waning theta–gamma-band coupled activity in specific topographic thalamocortical columns, resulting from auditory deafferentation (39). It can be conceived that the theta activity is the carrier wave connecting widespread areas (72), and that focal gamma-band activity in geographically separated brain areas is synchronized by nesting on the theta phase, as exemplified in auditory attention (106).

**Table 1:** **Summary of physiological and pathological oscillatory activity and cross-frequency coupling**.

		Physiological	Pathological
**Activity**	Theta	Carrier wave for memory-based information	Theta can be slowed alpha
	Alpha	Carrier wave for attentional processing	Alpha can be accelerated theta
	Beta	Information processing: reflects *status quo*, top-down prediction	Unknown
	Gamma	Information processing: reflects change, bottom-up prediction error, focal, waxes, and wanes	Persistent
**Cross-frequency coupling**			
	Thalamocortical	Alpha–gamma	Theta–gamma: slowing down alpha to theta permits access to parahippocampal auditory memory
	(Para)hippocampocortical	Theta–gamma	Alpha–gamma: accelerating theta to alpha permits attentional processing of tinnitus-related gamma activity

It has been suggested that in physiological circumstances alpha–gamma coupling may be related to thalamocortical circuits (107, 108), whereas theta–gamma coupling has been associated with fronto–hippocampal networks (34, 108). This suggests that the theta activity in TCD is actually slowed alpha, as originally proposed (39), since it is found in thalamocortical loops. We could further speculate that alpha (lagged) phase synchronization between parahippocampal- and subgenual cortex in tinnitus distress (47, 109) may in turn represent accelerated theta activity. Indeed, heart rate variability is controlled by theta activity in the subgenual anterior cingulate cortex (110), but in distress, alpha activity is noted in this same area (44, 47, 111, 112). As distress is linked to autonomic system changes (110, 113), this suggests that the alpha in distress could actually be high theta. Thus, the difference between physiological and pathological theta–gamma coupling might not only be the transient versus persistent activity but also the generator, i.e., thalamus versus (para)hippocampus.

From its inception, it was also proposed that TCD is not restricted to the auditory system, but involves both the medial and lateral thalamic system (39). The additional involvement of the context-processing, non-specific medial thalamic nuclei, which connect to the cingulate, medial prefrontal, and orbitofrontal cortices, would provide an explanation for the affective component changes (39) present in about 20% of all people with tinnitus (114). The insula is involved in tinnitus. The anterior insula and dorsal anterior cingulate are part of a salience network, which is activated in chronic tinnitus (86, 115–117). This could reflect a paradoxical salience attributed to the tinnitus sound (3, 46–48), a reason why it remains present at a conscious level. Indeed, the right insula has been linked not only to conscious processing of cognition and emotion (118, 119) but also to the perception of tinnitus distress, reflected by sympathetic hyperactivity (113). The insula is hypothesized to integrate interoceptive and exteroceptive salient information (120), generating a feeling (118) of risk (121) and meaning (122) to the sound. The tinnitus seems to be processed by the brain as exteroceptive information as remembering a sound deactivates the insula and anterior cingulate cortex (123). Even though this concept is plausible, experimental support has only been found in the insula in distressing tinnitus (113). Most oscillatory changes in this non-specific distress network are found to occur in the alpha-frequency domain (44, 47, 111), and not in theta- or gamma-band activity. But as speculated before, this could actually reflect another form of dysrhythmia, in which theta is increased to alpha, and linked to the septal nuclei, as generator of hippocampal theta, rather than the thalamus. Indeed, septal nuclei burst at theta frequencies and fire tonically at 10–15 Hz when depolarized (124).

## Thalamocortical Dysrhythmia and Error Prediction in Tinnitus

It has been proposed that tinnitus is an emergent property of network activity that fills in the deafferented thalamocortical activity in order to reduce auditory uncertainty (105). This goes back to the concept of the brain as a “prediction machine” as a fundamental characteristic of engagement with the world (125). The predictive brain requires an internal representation of the world (125), against which sensory stimuli are compared, and in which they are integrated. It incorporates the idea that a crucial function of the brain is to use stored information to imagine, simulate, and predict possible future events (126). The brain aims to minimize its prediction errors about its environment according to the free-energy principle (127). The Bayesian brain model adds to the predictive brain model that the predictions the brain makes are updated via active exploration of the environment through the senses (128). From a Bayesian statistical point of view, the brain is conceived as an inference machine that actively predicts and explains its sensations (127). Its central postulate is that percepts represent predictions or hypotheses about the external world whose degree of belief (the posterior probability) is determined by a combination of sensory evidence obtained by actively sampling the environment and background assumptions, based on a model of the world stored in contextual memory, about *a priori* plausible world structures (the prior) (14, 128). Subsequently, the posterior belief (=percept) becomes the next prior belief (=prediction) for a new sequence of prediction, resulting in a Markov chain Monte Carlo inference, i.e., a constantly updated hypothesis of the world. This requires some form of intentionality (129) related to self-preservation. Auditory deafferentation in a given frequency range induces a tonotopically specific prediction error. The actual auditory input is inconsistent with auditory input before the hearing loss occurred (3). In other words, the actual input is inconsistent with what is stored in memory. This inconsistency in turn signals that an information update is required (41). The oscillatory activity related to auditory predictions has been recently identified (130): delta–beta coupled oscillations underpin prediction accuracy (130), and (Bayesian) updating of the predictions is processed by the alpha-band (10–14 Hz) (130). Predicting “when” an auditory stimulus arrives predominantly involves low-frequency delta and theta oscillations, predicting “what” is processed by gamma and beta oscillations (131). Beta oscillations likely underlie a top-down flow of information, whereas gamma oscillations could be generated bottom-up (131). Whereas predictions are transmitted in a top-down “backward” manner, using mainly the beta band, prediction errors could be propagated in the gamma band in a bottom-up feed-forward manner (131). Updating of the predictions, via attention-based scanning of the environment (132), on the other hand, is linked to alpha oscillations (130, 132). Thus, transferring these findings to tinnitus, on could speculate that increased gamma activity in tinnitus would be related to a deafferentation-related (thalamocortical column specific spatial mismatch) prediction error, and the nesting on theta or delta related to its temporal prediction. In other words, gamma activity could reflect any change in the auditory environment, as this induces a prediction error. In TCD gamma activity would reflect a persistent deafferentation-based thalamocortical column specific prediction error.

One could interpret the decrease of alpha in TCD as a decrease in environmental scanning, resulting in a persistent prediction error, i.e., a persistent tinnitus percept. The slowed down alpha, resulting in theta bursting can then recruit other areas that also burst in theta such as the septal nuclei driven hippocampal–parahippocampal memory network. Thus, by decreasing its firing and oscillation rate to theta the TCD permits to access memory to fill in the missing auditory information.

In summary, the Bayesian model for tinnitus gives an alternative but complementary explanation for the oscillatory changes seen in tinnitus by adding that also other cross-frequency couplings need to be considered in the pathophysiology of tinnitus.

## Thalamocortical Dysrhythmia and Deficient Inhibitory Top-Down Mechanisms

Tinnitus was proposed to be a bottom-up generated TCD, linked to thalamocortical deafferentation (39). However, from the beginning of this model, it was conceived that top-down mechanisms may also contribute to the development of TCD. This would be more related to neuropsychiatric or affective behavioral changes (133). In a normally functioning noise-cancelling mechanism alpha activity, which is the normal resting state activity in the ventral medial prefrontal cortex–pregenual anterior cingulate cortex should predominate. In a dysfunctional noise-cancelling mechanism, it is expected that delta/theta and beta would predominate. Indeed, in a study looking at selective enriched acoustic stimulation, in which overcompensation of the deafferented stimuli was used, clinical worsening of patients was related to increased beta activity in the pregenual anterior cingulate cortex, linked to increased gamma in the auditory cortex (90). This is in agreement with a study that shows that tinnitus distress is related to high beta (25 Hz) in frontal areas (44, 134). By contrast, in a study using bifrontal tDCS, improvement of tinnitus loudness and distress was associated with a decrease in gamma-band activity in the auditory cortex, mediated via increased alpha activity in the pregenual anterior cingulate cortex (87). A recent EEG study demonstrated a correlation between delta, theta, and beta1 activity in the pregenual to rostral ACC and the percentage of the time that tinnitus is perceived (135). The percentage of the time tinnitus is perceived seems, furthermore, to be inversely related to the functional connectivity between this area and the left primary auditory cortex (135). Even though these studies provide certain support for the involvement of the pregenual anterior cingulate cortex in changing auditory cortex gamma-band activity linked to tinnitus, these findings have still to be considered as preliminary and the exact mechanism how the pregenual anterior cingulated interacts with the auditory system is not yet revealed.

## Thalamocortical Dysrhythmia in Tinnitus Can be Modulated

In a study evaluating electrophysiological changes associated with an improvement in tinnitus perception by means of auditory coordinated reset stimulation (136), pretreatment pathological delta and gamma-band activity was normalized only in those patients who had a benefit from the treatment (89), demonstrating that TCD is possibly causally related to the tinnitus.

MEG measurements before and after repetitive transcranial magnetic stimulation (rTMS) revealed that subjective reduction of tinnitus loudness after rTMS was related to a reduction of gamma and an increase in alpha activity (137).

Neurofeedback is a promising neuromodulation technique, based on operant conditioning, which can modulate TCD. It has been shown that tinnitus is related to a loss of alpha power, which is associated with an increase in gamma power (92), and that gamma is coupled to theta (84), the signature of TCD. Using neurofeedback in tinnitus, the alpha power can be increased in the auditory cortex (138), returning to a normal rhythmic alpha resting state.

Surgical neuromodulation treatments such as thalamic lesioning (52, 139) and auditory cortex stimulation via implanted electrodes (46, 93) have shown to reduce tinnitus in selected patients and to alter electrophysiological signatures of tinnitus-related TCD (46, 93, 139). In a tinnitus patient who was implanted with electrodes overlying the auditory cortex for tinnitus treatment, recordings from the implanted electrode revealed increased gamma and theta activity only at one single pole, whereas at the other electrode poles normal alpha activity was recorded, demonstrating a highly spatially specific TCD. Furthermore, autocorrelations demonstrated that the theta and gamma activity was coupled, giving further support to the concept of TCD. The pole with increased gamma and theta activity exactly co-localized with the hotspot of maximal blood oxygen level-dependent activation during presentation of the tinnitus tone in the fMRI scanner. Moreover, maximal tinnitus suppression was obtained when current was delivery exactly at that cortical area. Tinnitus suppression in turn was accompanied by normalization of theta and gamma spectral changes, as well as the disappearance of the coupled theta–gamma activity both on electrode and source-localized electroencephalography recordings (46). These TCD-related changes were not seen in the areas at a distance of the BOLD activation, and stimulation at the normal sites did not clinically benefit the patient.

These findings, which are still limited to a small number of cases, provide further support for a causal relationship between TCD and tinnitus.

The question arises why tinnitus is so stable, so rigid, and difficult to reverse. Even though no proven answer can be given a heuristic concept can be forwarded. For pain, it has been suggested that it should be conceived of as a homeostatic emotion (118), and tinnitus could be considered as a homeostatic emotion as well, a balance between mechanisms that increase sound processing and mechanisms that decrease sound processing (41). From a Bayesian predictive point of view, an allostatic mechanism may induce a reference resetting in chronic tinnitus, such that the tinnitus state becomes the norm instead of the silent state (48), analogous to what is described in addiction (140). There are currently no studies performed looking at oscillatory signatures for allostasis, but it can be theoretically expected that allostasis is mediated by altered high-frequency oscilliations in the pregenual anterior cingulate and dorsal ACC, resetting the balance between noise-cancelling mechanisms and areas that process loudness (99). Once the auditory homeostasis is fluctuating around a tinnitus state as reference or norm, it becomes more difficult to treat. This is confirmed in clinical data, where tinnitus becomes more difficult to treat by brain stimulation techniques such as TMS (141–143) or operative techniques (144–146).

## Is Thalamocortical Dysrhythmia Present in all Tinnitus Patients?

Based on its theoretical underpinnings TCD could be absent in tinnitus patients without deafferentation. However, it has to be considered that deafferentation does not equal behaviorally measurable hearing loss (147). Indeed, one can cut part of the auditory nerve without auditory threshold changes (148). Based on the Bayesian brain model, it has been theoretically proposed that depending on the bandwidth of deafferentation different compensatory mechanisms are utilized to obtain the missing auditory information, as to reduce auditory uncertainty related to the deprivation of auditory input (105).

**Figure 3:**
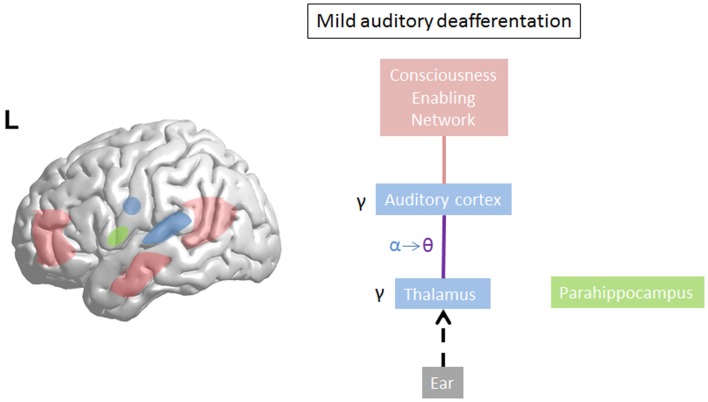
**On limited deafferentation thalamocortical resting state alpha activity slows down to theta activity resulting in a decreased surround inhibition, so that the missing auditory information can be retrieved from the cortical neighborhood (purple line)**. The tinnitus sound representation is encoded by gamma. The theta–gamma coupled activity is called thalamocortical dysrhythmia.

**Figure 4:**
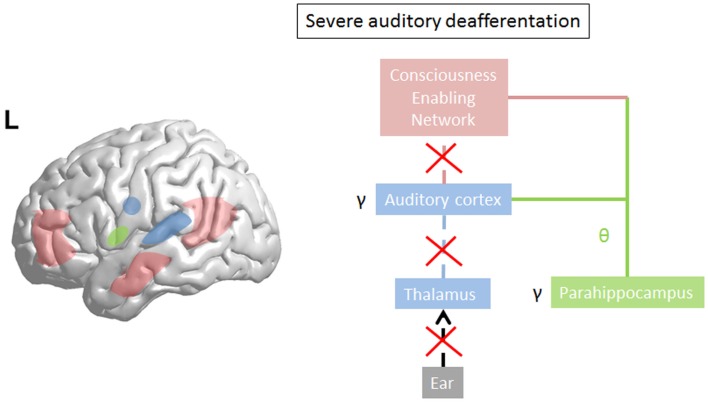
**In severe deafferentation, the auditory cortex cannot retrieve the missing auditory input from the (auditory) cortical neighborhood**. The thalamocortical bursting theta activity in the auditory cortex recruits the missing auditory input from the most recent update from parahippocampal-mediated memory processing (green line). There is no more thalamocortical theta–gamma dysrhythmia but a physiological memory related parahippocampocortical theta–gamma rhythm. The parahippocampus becomes linked to the consciousness enabling network (red line). Gamma decreases in the auditory cortex (arrow) as the prediction error disappears in comparison to what the parahippocampus predicts.

Limited damage to auditory receptors results in loss of functional surround inhibition in the cortex, causing unmasking of latent inputs and significantly altering neural coding. However, these changes do not lead to plasticity of the cortical map (149), as the missing information can be obtained via access of overlapping tuning curves of the neighboring cortical cells. This is in accordance with clinical data demonstrating that in patients without hearing loss, no map plasticity is noted on fMRI (150). This decreased surround inhibition is also seen in TCD, in which GABAa mediated decrease in surround inhibition is noted, surrounding the thalamocortical columns that oscillate in theta (85) (Figure 3). If the deafferentation covers a broader frequency range, the brain reacts with widening of auditory receptive fields (151), which permits to pull the missing information from the auditory cortical neighborhood. If this is not possible, due to a more extended deafferentation, dendritic and axonal rewiring can occur (152), still retrieving the missing input from the auditory cortex. If the deafferentation is too extensive for compensatory auditory cortical plasticity to fill in the missing information, it is proposed that the missing data are retrieved from auditory memory via a parahippocampal mechanism (13, 105) (Figure 4). From a clinical point of view, this suggests that tinnitus associated with mild deafferentation (<20 dB HL) should theoretically be treated by targeting the auditory cortex, whereas tinnitus with hearing loss (>20 dB) might benefit more from targeting the parahippocampal area. It needs to be mentioned that the exact cut-offs for the different compensation mechanisms (decrease in surround inhibition, widening of receptive fields, sprouting, memory retrieval) have not been investigated yet. This could also explain that when oscillatory brain activity is compared between responders and non-responders to implanted electrodes overlying the auditory cortex, the difference is in the parahippocampal area (beta3 and gamma), not in the auditory cortex, and that only those patients respond who have good functional connectivity between the auditory cortex (where the stimulating electrode is) and the parahippocampus (153). Clinically, the responders are also characterized by an exact match between the tinnitus frequency spectrum and the frequency of hearing loss, suggesting that their tinnitus is primarily caused by deafferentation (153). This suggests that in tinnitus with severe hearing loss the dysrhythmia could be centered on the parahippocampus rather than auditory cortex and thalamus. The parahippocampus’ involvement in tinnitus has been shown, both in rsfMRI (154, 155), EEG (44, 45, 47, 86, 87, 110, 111, 115, 116, 153, 156–160), SPECT (161), and PET (162, 163) studies. Parahippocampal functional interactions with auditory cortex, i.e., functional connectivity has been demonstrated both with resting state EEG (117, 153), MEG (164), and resting state fMRI (154, 155). In the parahippocampal area, 35% of cells respond to complex auditory stimuli (165) and 2% specifically and selectively to auditory stimuli (165). This area has been called the gatekeeper to the hippocampus (166), functioning as a sensory gate for incoming irrelevant or redundant auditory input (167). In other words, the posterior parahippocampal area can be considered as the main node of entry for auditory information to the medial temporal lobe memory system, where salient information is encoded into long-term memory (168). As the parahippocampal area has been hypothesized to play a central role in memory recollection, sending information from the hippocampus to the association areas, a dysfunction in this mechanism is posited as an explanation for complex auditory phantom percepts such as auditory hallucinations (169). It might therefore also be involved in the generation of simple auditory phantom percepts such as tinnitus (3). This could also explain why not in all patients with tinnitus increased auditory gamma-band activity can be detected (54) and why some studies do not find a correlation between the presence of tinnitus and gamma-band activity (94).

## Conclusion

Thalamocortical dysrhythmia is the consequence of hyperpolarization of the thalamus, i.e., by a disconnected thalamus, due to deafferentation. The deafferentation results in alpha activity to slow down to theta activity, i.e., in decreased information input from externally, but permits to obtain the missing information from the auditory cortex neighborhood due to decrease in surround inhibition. Mechanistically, this is mediated by de-inactivation of T-type Ca^2+^ channels, and the generation of low-threshold bursting, normally only present during sleep. However, in case of severe deafferentation, when the missing information cannot be found in the auditory cortex neighborhood, the bursting theta activity acts as a carrier wave to access (=synchronize) (para)hippocampal theta activity-related memory processes. Persisting deafferentation could result in attentional hyperactivity and increased salience (looking for missing input), possibly resulting in accelerating theta to alpha activity, associated with tinnitus distress. Thus, TCD can be considered as an adaptive mechanism to retrieve missing auditory input and gaining access to consciousness enabling networks in tinnitus. This is in accordance with the Bayesian brain concept that states that tinnitus is an attempt to decrease auditory deprivation related uncertainty. TCD might not be present in patients without deafferentation, as in this subgroup a noise-cancelling mechanism might underly the generation of tinnitus. The exact mechanism of how the noise-cancelling mechanism modulates thalamocortical activity is unknown.

Thalamocortical dysrhythmic cross-frequency coupled activity *per se* is not sufficient for conscious awareness of tinnitus, and neither is parahippocampocortical cross-frequency coupled activity. For it to reach consciousness, the cross-frequency coupled activity needs to be functionally coupled with a consciousness enabling network, which is mediated via the theta oscillation as a carrier wave for the percept and alpha for attentional processing. As such, tinnitus is an emergent property of auditory (mild deafferentation) or parahippocampal (severe deafferentation) gamma-band activity connected to a consciousness enabling network via low-frequency carrier waves. From a Bayesian point of view, the gamma activity of the TCD represents a bottom-up transmitted prediction error.

## Conflict of Interest Statement

The authors declare that the research was conducted in the absence of any commercial or financial relationships that could be construed as a potential conflict of interest.
